# Effectiveness of computer-tailored Smoking Cessation Advice in Primary Care (ESCAPE): a Randomised Trial

**DOI:** 10.1186/1745-6215-9-23

**Published:** 2008-04-29

**Authors:** Hazel Gilbert, Irwin Nazareth, Stephen Sutton, Richard Morris, Christine Godfrey

**Affiliations:** 1 Department of Primary Care and Population Sciences, Royal Free and University College Medical School, London, UK; 2 GP Principal, The Keats Group Practice, 1b Downshire Hill, London, UK; 3 Institute of Public Health, University of Cambridge, Cambridge, UK; 4 Department of Health Sciences, University of York, York, UK

## Abstract

**Background:**

Smoking remains a major public health problem; developing effective interventions to encourage more quit attempts, and to improve the success rate of self-quit attempts, is essential to reduce the numbers of people who smoke. Interventions for smoking cessation can be characterised in two extremes: the intensive face-to face therapy of the clinical approach, and large-scale, public health interventions and policy initiatives. Computer-based systems offer a method for generating highly tailored behavioural feedback letters, and can bridge the gap between these two extremes. Proactive mailing and recruitment can also serve as a prompt to motivate smokers to make quit attempts or to seek more intensive help. The aim of this study is to evaluate the effect of personally tailored feedback reports, sent to smokers identified from general practitioners lists on quit rates and quitting activity. The trial uses a modified version of a computer-based system developed by two of the authors to generate individually tailored feedback reports.

**Method:**

A random sample of cigarette smokers, aged between 18 and 65, identified from GP records at a representative selection of practices registered with the GPRF are sent a questionnaire. Smokers returning the questionnaire are randomly allocated to a control group to receive usual care and standard information, or to an intervention group to receive usual care and standard information plus tailored feedback reports. Smoking status and cognitive change will be assessed by postal questionnaire at 6-months.

**Discussion:**

Computer tailored personal feedback, adapted to reading levels and motivation to quit, is a simple and inexpensive intervention which could be widely replicated and delivered cost effectively to a large proportion of the smoking population. Given its recruitment potential, a modest success rate could have a large effect on public health. The intervention also fits into the broader scope of tobacco control, by prompting more quit attempts, and increasing referrals to specialised services. The provision of this option to smokers in primary care can complement existing services, and work synergistically with other measures to produce more quitters and reduce the prevalence of smoking in the UK.

**Trial registration:**

Current Controlled Trials ISRCTN05385712

## Background

Smoking remains a major public health problem; 23% of all deaths in middle age are attributed to smoking [1]. Mortality from tobacco in the first half of the 21^st ^century will be affected much more by the number of adult smokers who stop than by the number of adolescents who start [2]. Declines in smoking rates have been greater among the better educated, leading to a strong socio-economic gradient in smoking prevalence [3] contributing to health inequalities and accounting for over half of the difference in risk of premature death between the social classes [4]. Developing effective interventions to encourage more quit attempts, and to improve the success rate of self-quit attempts, is essential to reduce the numbers of people who smoke, and to reach the Department of Health's targets to reduce the prevalence of smoking in manual groups from 32% to 26% over the next eight years [5].

Smoking cessation services are currently high on the NHS agenda, with strong drive to deliver a variety of interventions in primary care. Interventions for smoking cessation can be characterised in two extremes. The treatment model of the NHS services is based on the clinical approach, offering intensive support and focusing on individual face-to-face treatment. As a result of government initiatives [6], the number of specialist clinics offering intensive face-to-face treatment for smoking cessation, which can produce relatively high abstinence rates, has increased. However, they are limited by low participation rates, less than 2% of smokers make use of these specialist services [7-9]. At the other extreme are large-scale, public health interventions and policy initiatives (e.g. legislation to control smoking in public places). This type of intervention can reach large numbers of smokers, but effectiveness rates are low partly because such interventions lack the personal element that is central to the success rates of the clinical approach. Computer-based systems offer a method for generating highly tailored behavioural feedback letters [10], which can be cost-effectively produced on a large scale, with the potential to help a greater proportion of the smoking population in changing their behaviour. The majority of smokers prefer less intensive methods [11] and tailored self-help materials can bridge the gap between these two extremes, combining the behavioural intervention principles used in clinical interventions with the participation rates of public health interventions. Furthermore, as many as 70% of smokers have no serious intentions to attempt to quit in the next year [12,13]. Proactive mailing and recruitment outside the general practice offering personalised service, as an alternative or addition to brief intervention delivered by GPs, could also increase the impact of legislation, by serving as a prompt to motivate smokers to make quit attempts or to seek more intensive help through NHS services.

A large number of studies of tailored feedback have been published demonstrating positive results. This trial uses a modified version of a computer-based system developed by two of the authors (HG and SS) to generate individually tailored feedback reports designed to encourage and help smokers to quit. The smoker first completes a paper-based Smoking Behaviour Questionnaire (SBQ). The SBQ data are scanned into a database, and the computer program generates a feedback letter that is highly tailored to the individual's responses.

The efficacy of our tailored feedback was evaluated as an adjunct to telephone counselling (via the national Quitline) in a randomised trial of 1508 participants [14]. Results showed that at the 6-month follow-up, for the majority who were smokers at baseline (*n *= 1164), this intervention significantly increased 1-month prolonged abstinence rates from 11.3% in the control group to 16.4% in the intervention group (p < 0.02). These findings are consistent with previous studies, which have demonstrated a positive effect of individually tailored printed self-help material on smoking cessation [15-17], and suggest that these effects may increase with time [9], in contrast to clinic based treatments which have large effects immediately post treatment but decline over time [18,19]. A Cochrane review [20] reported on a meta-analysis of seventeen trials using materials tailored to the characteristics of individual smokers. While part of the effect could be due to the additional contact or assessment required to obtain individual data, there is evidence that tailored materials increase quit rates over and above standard materials and untailored materials (OR 1.42, 95% CI 1.26 – 1.61).

Two exceptions to this are recent UK general practice based studies that failed to demonstrate an increase in cessation rates for tailored feedback compared with an untailored letter [21] or with standard self help literature [22]. Both were based on the transtheoretical model (TTM) [23] which has serious conceptual and measurement problems [24,25]. The materials used in the study by Lennox and colleagues were also lacking in the depth of individual tailoring and in practical advice on how to stop smoking [26]. Our tailored intervention differs from that used in these two studies. Development of our intervention was informed by evidence from the smoking cessation literature, using specific concepts from different theoretical models of behaviour change that have been shown to be relevant to change. It aims to change the cognitive determinants of smoking and smoking cessation, by addressing beliefs and expectations, and attitudinal ambivalence towards quitting, by enhancing perceived self-efficacy and re-evaluating the social environment. Theoretically, this is consistent with a number of social cognition models that have been applied to understanding smoking cessation. Previous research [15] has suggested the importance of tailoring to specific theoretical constructs. Matching advice to stage of readiness is common in many of the existing computerised programs. However, the use of one model exclusively, e.g. the transtheoretical or "stages of change" model (TTM) [23], to drive the tailoring does not allow for individual variation from that pattern of change or in the sequence of movement along the continuum. Stronger tailoring programs can be produced by using multiple theories to inform the tailoring process [27]. In addition to this strong theoretical base, our system is based on empirical findings (e.g. on predictors of quit attempts and success). By identifying specific factors that influence behaviour and decisions, we offer information to encourage individuals to bring about the desired cognitive states or behaviour for successful quitting. Moreover, the feedback reports were developed in consultation with smoking cessation counsellors and include conventional wisdom (e.g. the importance of setting a quit date). Our tailored feedback reports are therefore more psychologically detailed than those used previously in UK studies. The format resembles a personal letter, and differs from the promotional leaflet style used in other studies.

Since our previous study with the Quitline, [14] we have done extensive work to develop the intervention, consistent with the MRC Framework for the Development and Evaluation of Complex Interventions [28].

Smoking cessation literature has been criticised in the past for being written at a level beyond the literacy skills of many smokers [29], leading to higher levels of education associated with the use of self-help materials [16]. Results from our previous study [14] suggest that the tailored feedback may be as effective among more deprived smokers as in the less deprived. Extensive work on the development of the intervention, in consultation with literacy experts, has modified the questionnaire and tailored the format and content of the advice to different educational levels. Focus groups engaged users (i.e. smokers) in exploring the acceptability and suitability of the materials, and their suggestions incorporated into the modifications.

In addition, the acceptability and feasibility of the delivery of this modified feedback in general practice using a proactive recruitment strategy has been assessed in a pilot trial [30]. Questionnaires were sent to a random sample of 200 smokers identified from GP records from each of four practices. Results indicate that a 10% response rate can be achieved with one mailing. There are strategies that can be used to increase response rates [31], and by using some of these strategies (e.g. a reminder with a second copy of the questionnaire, a more attractive questionnaire with the use of colour) we estimate that the response rate could be increased to 15%.

The results of the pilot trial also indicate that a high proportion of the respondents are not planning to quit in the next six months [30]. It is essential that we develop methods to engage these smokers in cognitive change to encourage modification of attitudes and beliefs to promote cessation activity in this population group. To address this we have incorporated cognitive strategies and motivational interviewing techniques which are generally more appropriate for smokers not ready to quit [32], into the tailored interventions in varying degrees to suit the motivation of the smoker. In our previous study of callers to the Quitline [14], participants received a single feedback letter. However, this system can store data, to be used at a later date in combination with new information provided by the smoker. In the present study the data is being used in this way to expand the intervention by including a second assessment and letter. Multiple tailoring has been shown to be more effective than single tailoring in promoting cognitive change in smokers with low readiness to quit [33].

The aim of this study is to test the hypothesis that personally tailored feedback reports, based on an assessment of individual needs and tailored to levels of reading ability, sent to smokers identified from general practitioners lists with varying levels of motivation and readiness to quit, will increase quit rates and quitting activity over and above that found with standard self help and usual care received from the practice. The study objectives are 1) to compare the effectiveness of sending personalised computer tailored feedback reports to smokers with sending standard self-help materials 2) to explore the effectiveness of tailored feedback reports by socio-economic status to determine their effect in more deprived groups 3) to determine the characteristics of smokers who are prompted to change their behaviour after receiving tailored feedback reports.

## Methods

### Design of the study

The study is a randomised controlled trial of cigarette smokers identified from GP records and sent a questionnaire. Smokers returning the questionnaire are randomly allocated to a Control Group, to receive standard help, or to an Intervention Group to receive tailored feedback reports. The trial is conducted as a collaboration between UCL and University of Cambridge, and is co-ordinated from UCL. The study has been reviewed and approved by the Northern and Yorkshire MREC, and has received R&D approval from all participating PCTs.

The research has substantial potential to benefit participants, by improving health through a positive change of lifestyle. The information leaflet also informs participants that while there is no guarantee that the information they receive will help, they may find that they learn more about themselves by answering the questions. The aim of the intervention is to help participants to stop smoking, it is therefore unlikely that there will be any adverse effects. However, anyone experiencing distress as a result of the assessment or intervention, is advised to discuss it with their GP or a counsellor.

### Participants and recruitment

The MRC General Practice Research Framework (GPRF) is a network of 1100 practices scattered throughout the UK. We are recruiting 100 GPRF practices, selected to represent high and low socio-economic areas to maximise the generalisability of the results. Practices generally identify 13% to 22% of their patients as smokers, depending on the characteristics of the patient population, and the accuracy and completeness of the records [30]. A medium sized practice with 6000 to 7000 patients, would therefore identify between 780 and 1540 smokers. A sample, randomly selected using the practice computer systems, of 500 smokers from each practice (a total of 50000) are sent an assessment questionnaire, together with a covering letter from their GP. Using proactive recruitment to contact smokers directly and invite them to participate, we aim to secure a return rate in the order 15%, from 2 mailings based on previous studies [17,21], and on our pilot study to secure 7250 participants over a 12 months period from August 2007 to July 2008.

### Inclusion/exclusion criteria

All current cigarette smokers aged 18 to 65 able to read English are eligible for inclusion in the study. Exclusion criteria are minimal because the aim is to recruit all smokers. However, any patients selected who are considered by the GP to be unsuitable for the project, e.g. people with severe mental impairment or severely or terminally ill, are excluded.

### Randomization

Those willing to be contacted for future assessment return the completed questionnaire, together with a signed consent form, to the research team at University College London. Randomisation is at the level of the study participants. Upon receipt of the completed questionnaire eligible participants are randomly assigned to one of the two conditions, according to an externally constructed randomisation plan. The general practices represent a clustering variable and to reduce any confounding by practice we are using block randomisation, using blocks of eight (within which the order of assignment will be random), within each practice to allocate patients to each condition to closely balance the numbers. In a large trial such as this, we expect the conditions to be balanced on important predictive factors such as dependence.

However, any imbalances will be controlled for in the statistical analysis. While it is not possible to blind participants to the receipt of a feedback letter, in order to ensure that the researchers in all cases are blind to the allocation of the participant, the feedback is generated by a service administrator. To avoid bias in the outcome assessment, when conducting follow-up interviews by telephone for non-responders the interviewer will be blinded to the allocation of the respondent.

### Trial interventions

Participants are sent materials according to their randomisation, immediately after returning the questionnaire. Participants allocated to the Control Group are sent standard non-tailored information (the NHS 'SMOKEFREE' booklet), as well as receiving the usual care offered by their general practice. To eliminate any variation in the care offered, usual care is defined from the results of the pilot study, and we attempt to ensure that all recruited practices offer the same protocol. Participants allocated to the Intervention Group are sent a computer-tailored feedback report based on the information obtained at baseline, in addition to the standard non-tailored information and receipt of the usual care offered by their general practice. The Intervention Group are also sent an additional assessment to generate a further personal progress report one-month after the baseline.

### Materials

The baseline assessment questionnaire (SBQ) assesses demographic characteristics (including educational and literacy level), intention to quit, motivation, dependence, previous quit attempts, perceived advantages and disadvantages of quitting, self-efficacy and social environment. The baseline data are scanned and entered using FORMIC data capture software, simultaneously creating an SPSS file containing data for analysis, and a data file for producing feedback letters. A computer program temporarily combines these data with the personal data of participants allocated to the Intervention Group, selecting the correct messages from the message library to produce a feedback report. Once the report is produced the file is deleted, in accordance with the Data Protection Act. Participants are informed of this possible use of the data.

### Time schedule

Participants in the Control group receive the standard self-help immediately after returning the questionnaire. Participants in the Intervention group also receive the additional help in the form of a personal feedback letter immediately after returning the questionnaire, and a further one-month assessment for generating a personal progress report. The time schedule for the mailing of assessment and follow-up questionnaires, and intervention materials is presented in Table 1.

**Table 1:** Time schedule for mailing of assessment and follow -up questionnaires and intervention materials

T1 = Baseline	T2 = 1 month	T3 = 6 months
Baseline questionnaire (SBQ) and GP covering letter sent from practice and returned to research team.	One-month assessment for generating a progress report, sent by research staff to Intervention Group participants re-assessing baseline measures.	Follow-up questionnaire sent by research team to all participants assessing:
Control and Intervention materials dispatched immediately.		Primary outcome
		Secondary outcome
		Process measures

### Outcome measures

Outcomes are to be measured by postal questionnaire. Non-respondents to questionnaires will receive one postal reminder. Non-respondents to the reminder will be contacted by telephone. In order to estimate the accuracy of self-reports, a random sample of 20% of the participants who report abstinence will have their status validated by salivary cotinine sample, obtained by post.

Long term abstinence has been thought of as the gold standard for evaluating smoking cessation interventions, but has been criticized as failing to measure other possible benefits of treatment, such as repeated quit attempts and shorter periods of abstinence [34]. Recent recommendations suggest the use of less stringent criteria of 4-week or 7-day abstinence [35]. Cessation-induction trials test a treatment to prompt cessation among all smokers, including those with low motivation [36], it is important therefore in such trials to measure cognitive change, which can lead to quit attempts, short periods of abstinence, and eventual prolonged abstinence. Six months is widely recommended as the minimum length of follow-up in such trials, and accepted by the Cochrane reviews on smoking cessation.

The Primary Outcome Measure is Prolonged abstinence for 1 month and for 3 months at the 6-month follow-up.

Secondary Measures are 24 hr and 7 day point-prevalence abstinence, quit attempts, changes in motivation and intention to quit, and in cognitions measured at baseline, use of NRT or Zyban, and any contact with advice services or health professionals (group, clinic, telephone, or face-to-face), use of NHS resources and other smoking cessation aids for economic analysis.

Process measures are adherence to advice, perceptions of the feedback reports, perceived personal relevance of the feedback reports.

Performance figures against target figures set by PCTs before and after the intervention will be obtained from General Practices as indicators of activity.

### Data analysis

#### Sample size and power calculations

Spontaneous cessation is difficult to estimate, but population cessation rates suggest that it is between 0.5% and 3% [3]. Studies aimed exclusively at self-quitting unaided attempts have found 6 month abstinent rates of 3% [37] and 4.9% [38]. Lancaster and Stead, in a review of thirteen trials comparing standard self-help with no help [20] found an average 6 month quit rate in the control groups of 5%, with the pooled intervention rate slightly higher. Based on these figures we might expect a 3 month abstinence rate in a control group receiving standard materials to be at the upper limit of estimates of spontaneous long-term abstinence in the population (i.e. 3%).

The Cochrane review [20] of seventeen randomised trials of smoking cessation found that individually tailored materials were more effective than standard and untailored materials (OR 1.42, 95% CI 1.26 – 1.61) where the main outcome was abstinence from smoking after at least 6 months follow-up. The authors report different odds ratios for studies grouped by type, and the range of estimates demonstrate the uncertainty in this area and the need for further research. A range of possible effect sizes that could be used to power the trial are presented in Table 2. To carry out the trial within the resources available, we aim to recruit a sample of 7250. While a sample of this size does not give adequate power at the lower limit, it will give the minimum power required to detect a difference using the midpoint of the range of odds ratios, assuming a two-tailed test and alpha of 0.05 [39].

**Table 2:** Effect sizes and power for different size samples

OR	% increase in cessation rate	Sample	% power
Lower 1.26	3.75	4350	25
		5800	33
		7250	40
		8700	47

Middle 1.42	4.21	4350	54
		5800	67
		7250	77
		8700	84

Upper 1.61	4.74	4350	83
		5800	92
		7250	97
		8700	99

Assuming a cessation rate of 3% in the control group, with the relative increases in the proportion who report continuous abstinence of 3 months or more at a 6-month follow-up in the intervention group, applying the lower, middle, and upper odds ratios of 1.26, 1.42, and 1.61 (and assuming that participants who cannot be contacted at follow up are still smoking)

#### Attrition and Compliance

Based on our previous studies, we expect approximately 20–25% attrition [14,40]. For the main analysis participants who cannot be contacted at follow-up will be assumed to be smoking (intention-to-treat analysis). Previous studies have found that the number of participants in the intervention group who recalled receiving a personal letter at the 6-month follow-up ranged from 64% [21] to 89% [17]. Analysis of the treatment effect will be based on the whole sample.

#### Planned analyses

Chi-squared tests will compare binary outcomes between the intervention and control groups (e.g. for prolonged abstinence of 4 weeks), with logistic regression to take into account any chance imbalance in important baseline characteristics between the groups. Continuous variables (e.g. cognitive changes) will be compared with the two-sample t-test, with multiple regression to account for other important characteristics. Odds ratios and 95% confidence intervals for differences in means or medians (as appropriate) will be quoted.

To address drop-out, the primary analysis will assume that non-responders continue to smoke. Multiple imputation methods will be used as a sensitivity analysis, where predictors of drop-out will be used to impute likely outcomes (SOLAS software).

Because benefits of interventions may differ by general practice, and patients within practices may demonstrate more similarity in their outcome than patients in different practices, multi-level models will be applied to take into account general practice effects (MLwiN software).

Participants will be categorised by socio-economic status, social deprivation and different educational level to explore the effectiveness of the intervention in these groups.

#### Economic and Quality of Life issues

The incremental cost-effectiveness of the individual feedback compared to the control condition will be assessed from the NHS perspective in line with methods recommended by NICE [41]. The costs of administration and generation of the feedback will be estimated along with the basic care package from the application of the trial protocols in the different practice settings. The follow-up questionnaire will ask participants about their use of NHS resources, additional primary care appointments or prescriptions and use of specialist smoking cessation services, and other cessation aids. The data on use from these questionnaires will be combined with national estimates of their long run marginal costs from a variety of sources. Six month quit rate (adjusted for cotinine validation) will be used with appropriate epidemiological and economic modelling techniques to estimate the potential quality of life years gained and future savings in smoking related health care costs. The primary analysis with extensive sensitivity analysis including bootstrapping and the use of cost effectiveness acceptability curves will be to assess the cost effectiveness of the intervention compared to the control. However, the data from the trial will also be used to model the overall immediate costs of implementing the intervention in GP practices (including any increased use of other smoking related services) for different types of practices (e.g. in high and low deprived areas) and for England as a whole. Modelling will also be undertaken of the population levels effects on the health of the population and the NHS (including allowance for future savings in NHS costs) and how this intervention may interact with other policy initiatives.

In accordance with NICE technical appraisal guidelines [41], we are using EQ-5D at baseline and at follow-up to investigate short-term quality of life changes. Quality and quantity of life issues will be addressed as part of further modelling of longer-term improvement effects.

### Consumer Involvement

Small groups of smokers recruited via the Quitline commented on the original questionnaire and feedback letter, which were modified accordingly. The smoking cessation counsellors at the Quitline also provided input. Ongoing research using Focus Group discussions with smokers have explored consumers' views on the acceptability of this approach to encourage and support smoking cessation. Follow-up questionnaires sent to all participants in the study will evaluate users' perception of the materials. Participants will have the opportunity to request a report of the results. A lay summary will be prepared and sent to all participants requesting one.

## Discussion

In the UK there are 450 new smokers every day [42]. At this rate of recruitment to the smoking population, there is an urgent need to develop and deliver cost-effective interventions to the bulk of the population, rather than to the minority of dependent smokers who seek specialized help [43].

It is important to offer a range of interventions that appeal to different individual needs and preferences [44]. Computer tailored personal feedback, adapted to reading levels and motivation to quit, is a simple and inexpensive intervention which could be widely replicated and delivered cost effectively to a large proportion of the smoking population. The current portfolio of NHS services does not include a highly tailored approach for smokers who do not wish to attend clinics or have face-to-face counselling.

Reaching and changing the behaviour of smokers, particularly heavily dependent smokers and those from lower socio-economic groups is a challenging task, and the effect of any intervention likely to be small. However, contrary to common assumptions, the proportion of hardcore smokers (defined by cigarette consumption and time from waking to first cigarette), forms a lower proportion of all smokers than in 1996 [45]. Although, the prevalence of smoking by socio-economic group remains unchanged, there are no differences by socio-economic group or by cigarette consumption in intentions and desire to quit and in attempts to quit [45], suggesting that these harder to reach groups will be as receptive to help and encouragement as higher socio-economic groups and less dependent smokers. Furthermore, a modest success rate could have a large effect on public health given its recruitment potential, and make a valuable contribution to lowering smoking prevalence.

Brief advice from GPs has been shown to increase quit rates [46], and this method enables the standardised collection of relevant information from smokers by practice nurses or other health professionals, and could offer an efficient tool to integrate smoking cessation counselling into a busy primary care practice. The degree of uptake by GPs of this intervention is an important issue requiring research in its own right. However, feedback from general practices will inform the degree of uptake as well as reasons for agreeing or refusing to take part in the study, and also identify any potential barriers to the implementation of the intervention. The provision of this option to smokers in primary care could complement existing services, and work synergistically with other measures to produce more quitters and reduce the prevalence of smoking in the UK. This is also an intervention that would fit into the broader scope of tobacco control, by prompting more quit attempts, and increasing referrals to specialised services.

## Abbreviations

EQ-5D: EuroQol 5D; ESCAPE: Effectiveness of computer-tailored Smoking Cessation Advice in Primary Care; GP: General practitioner; GPRF: General Practice Research Framework; MLwiN: Multilevel modelling for windows; MRC: Medical Research Council; MREC: Main Research Ethics Committee; NHS: National Health Service; NICE: National Institute for Clinical Excellence; NRT: Nicotine replacement therapy; PCT: Primary Care Trust; R&D: Research and Development; SBQ: Smoking Behaviour Questionnaire; SOLAS: Software Tool for Analyzing Incomplete Data; SPSS: Statistical Package for the Social Sciences; TTM: Transtheoretical model; UCL: University College London; UK: United Kingdom.

## Competing interests

The authors declare that they have no competing interests.

## Authors' contributions

HG conceived of the study, was involved in the development of the intervention and questionnaires, drafted the protocol and is the principal investigator.

SS is a co-investigator, and was involved in the conception and design of the study, in the development and adaptation of the intervention, and contributed to the writing of the protocol.

IN is a co-investigator with expertise in primary care research, was involved in the design of the study, contributed to the writing of the protocol, and is advising on the management and co-ordination of the trial, particularly the liaison with the GPRF.

RM is a co-investigator and trial statistician, and contributed to the protocol in matters relating to randomisation and statistical analysis.

CG is a collaborator, has commented on the protocol and contributed to matters relating to the analysis and reporting of economic and quality of life issues.

All authors read and approved the final protocol.
